# Natural Extracts in Skin Repair and Wound Healing: Phytochemical Mechanisms and Dermopharmaceutical Perspectives

**DOI:** 10.3390/molecules31060967

**Published:** 2026-03-13

**Authors:** Niki Tertipi, Vasiliki Sofia Grech, Eleni Sfyri, Eleni Andreou, Vasiliki Kefala, Efstathios Rallis

**Affiliations:** Department of Biomedical Sciences, School of Health and Care Sciences, University of West Attica, Campus 1, 12243 Athens, Greece; vgkrek@uniwa.gr (V.S.G.); elsfiri@uniwa.gr (E.S.); elandreou@uniwa.gr (E.A.); valiakef@uniwa.gr (V.K.); erallis@uniwa.gr (E.R.)

**Keywords:** wound healing, phytochemicals, natural extracts, molecular signalling pathways, polyphenols, redox signalling, inflammatory pathways, angiogenesis, dermopharmaceutical formulations

## Abstract

Background: Skin repair and skin wound healing are tightly regulated biological processes that require coordinated control of inflammation, redox homeostasis, angiogenesis, and tissue remodelling. In this context, natural extracts are increasingly recognized as sources of chemically diverse phytochemicals capable of modulating defined molecular signalling pathways that govern cutaneous repair. Methods: This review provides a mechanism-informed synthesis of current evidence by examining representative botanical sources, including *Aloe vera*, *Centella asiatica*, *Curcuma longa*, *Calendula officinalis*, and *Panax ginseng*, which have been extensively investigated in preclinical wound-healing models. Rather than providing an exhaustive catalogue of plant species or individual compounds, the analysis emphasizes how distinct phytochemical classes interact with conserved molecular pathways involved in skin repair. Results: Flavonoids, terpenoids, phenolic acids, alkaloids, and polysaccharides influence inflammatory signalling pathways, redox-sensitive pathways, growth factor-mediated responses, and cellular migration, thereby supporting phase-appropriate progression of wound healing. Recurrent modulation of NF-κB, TGF-β, VEGF, and Nrf2 signalling emerges as a central mechanistic theme. Advances in dermopharmaceutical formulation strategies, including hydrogels and lipid-based carriers, may enhance local delivery and stability of phytochemicals; however, their translational value remains dependent on chemical standardization and mechanistic validation. Conclusions: This review provides a mechanism-informed synthesis of current evidence, highlighting how phytochemical diversity, molecular signalling pathways, and dermopharmaceutical formulation strategies collectively shape the therapeutic potential of plant-derived extracts in cutaneous wound healing and may guide future mechanistic and translational research in phytochemical-based wound therapeutics.

## 1. Introduction

Effective skin repair requires tightly regulated interactions among inflammatory resolution, redox homeostasis, angiogenesis, and tissue remodelling. These processes must unfold within a precisely coordinated temporal sequence to achieve functional regeneration [1]. Therapeutic interventions that disrupt this balance, either through insufficient modulation or excessive and prolonged pathway activation, may delay wound closure, compromise tissue architecture, or promote fibrotic remodelling [2]. From a pharmaceutical perspective, the central challenge in wound management therefore lies not in the indiscriminate stimulation of repair pathways, but in their selective and temporally controlled modulation in alignment with the dynamic biology of the healing wound [3]. This perspective emphasizes the importance of pathway-specific and temporally coordinated therapeutic strategies rather than the generalized stimulation of tissue repair processes.

Within this mechanistic framework, natural extracts are increasingly investigated as sources of structurally diverse phytochemicals. These compounds are capable of interacting with defined molecular signalling networks rather than acting as nonspecific bioactive mixtures [4]. Experimental evidence increasingly indicates that phytochemicals modulate inflammatory signalling, growth factor-mediated responses, redox-sensitive pathways, and cellular migration in a context and phase-dependent manner [4]. However, the multifunctionality underlying these effects, characterized by pleiotropic molecular targets, compositional complexity, and sensitivity to dose and timing, also complicates mechanistic interpretation and translational extrapolation [5]. In addition to modulating inflammation and oxidative stress pathways, several plant-derived extracts exhibit antimicrobial activity against wound-associated pathogens and have been reported to influence extracellular matrix deposition, including collagen synthesis, in preclinical models. Without rigorous mechanistic resolution and appropriate pharmaceutical contextualization, such biological observations risk remaining correlative, particularly when derived from simplified experimental systems [5]. Consequently, distinguishing mechanistically relevant pathway modulation from nonspecific bioactivity remains a central challenge in the interpretation of phytochemical effects on wound repair.

A further dimension of complexity arises at the level of formulation science [6]. Many phytochemicals exhibit limited solubility, chemical instability, or restricted cutaneous penetration, necessitating delivery strategies that may substantially alter local bioavailability and pathway engagement. Although advanced dermopharmaceutical systems can mitigate some of these constraints, they introduce additional variables related to pharmacokinetics, pharmacodynamics, safety, and regulatory feasibility [6]. Accordingly, meaningful evaluation of plant-derived extracts in wound healing requires integrated consideration of molecular mechanisms, phytochemical composition, formulation design, and functional repair outcomes, rather than isolated assessment of biological activity or technological sophistication [6].

In recent years, several reviews have examined the biological and dermatological relevance of plant-derived compounds, particularly polyphenols, in skin physiology and pathology. Recent studies have explored the molecular mechanisms through which polyphenols influence skin ageing and cellular stress responses, highlighting antioxidant activity and modulation of inflammatory signalling pathways [7]. Other reviews have emphasized natural polyphenols as multifunctional bioactive compounds in dermatological and cosmetic applications, focusing on their anti-inflammatory, antioxidant, and photoprotective properties [8]. In addition, the literature has explored the potential role of these compounds in various skin disorders associated with oxidative stress and inflammatory dysregulation [9]. While these contributions provide valuable summaries of the dermatological relevance of polyphenolic compounds, they primarily emphasize descriptive accounts of biological activities or dermatological applications. They rarely integrate phytochemical composition, molecular signalling mechanisms, and pharmaceutical formulation considerations within the dynamic and phase-dependent context of wound healing.

Despite the growing body of literature addressing natural compounds in dermatology [7,8,9] an important conceptual gap remains between descriptive summaries of plant-derived bioactives and mechanistically resolved analyses of how distinct phytochemical classes regulate the coordinated signalling networks governing cutaneous repair. To our knowledge, relatively few reviews integrate molecular pathway regulation, phytochemical diversity, and dermopharmaceutical formulation strategies within a unified framework that reflects the temporal and cellular complexity of wound healing [7,8,9]. The present review addresses this gap by adopting a mechanism-driven and pharmaceutical-oriented perspective on botanical extracts involved in skin repair. Rather than cataloguing plant species or individual compounds, the review focuses on how structurally diverse phytochemical classes converge on conserved molecular signalling pathways regulating inflammation, redox homeostasis, angiogenesis, cellular migration, and tissue remodelling during skin wound healing. Representative medicinal plants are examined as mechanistic models linking phytochemical composition to pathway-specific biological responses. Dermopharmaceutical formulation strategies are discussed in relation to physicochemical constraints, bioavailability limitations, and translational feasibility. Addressing this conceptual gap is essential for translating research on plant bioactives from descriptive biological observations toward mechanistically grounded therapeutic strategies. Importantly, the present review differs from previous summaries by integrating phytochemical diversity, pathway-specific molecular mechanisms, and dermopharmaceutical formulation considerations within the dynamic and phase-specific biology of wound healing.

This review adopts a mechanism-oriented perspective on plant-derived extracts involved in skin repair and wound healing, emphasizing the convergence of phytochemical diversity, molecular signalling pathways and dermopharmaceutical formulation strategies. Rather than cataloguing plant species or individual compounds, the analysis focuses on how structurally diverse phytochemical classes converge on conserved molecular signalling pathways regulating inflammation, redox homeostasis, angiogenesis, cellular migration, and tissue remodelling. The review progresses from molecular mechanisms of phytochemical action to dermopharmaceutical formulation considerations and representative medicinal plant models, followed by a critical appraisal of current limitations and translational challenges in phytochemical-based wound therapeutics. By integrating phytochemical diversity, signalling pathway modulation, and dermopharmaceutical formulation strategies within the phase-specific biology of wound healing, this review provides a structured mechanistic framework that highlights the translational relevance of phytochemical-based interventions in skin repair.

## 2. Materials and Methods

Literature relevant to the molecular and pharmaceutical aspects of skin repair and wound healing was identified through targeted searches of PubMed, Web of Science, and Scopus. The primary focus was placed on studies published within the last 5–7 years to reflect current mechanistic understanding, while earlier publications were considered when they provided seminal insights into key signalling pathways or experimental models. Selection was restricted to peer-reviewed primary research articles addressing cutaneous wound healing, with emphasis on in vivo studies or in vitro investigations supported by a clear mechanistic rationale and demonstrable functional relevance.

**Figure 1:**
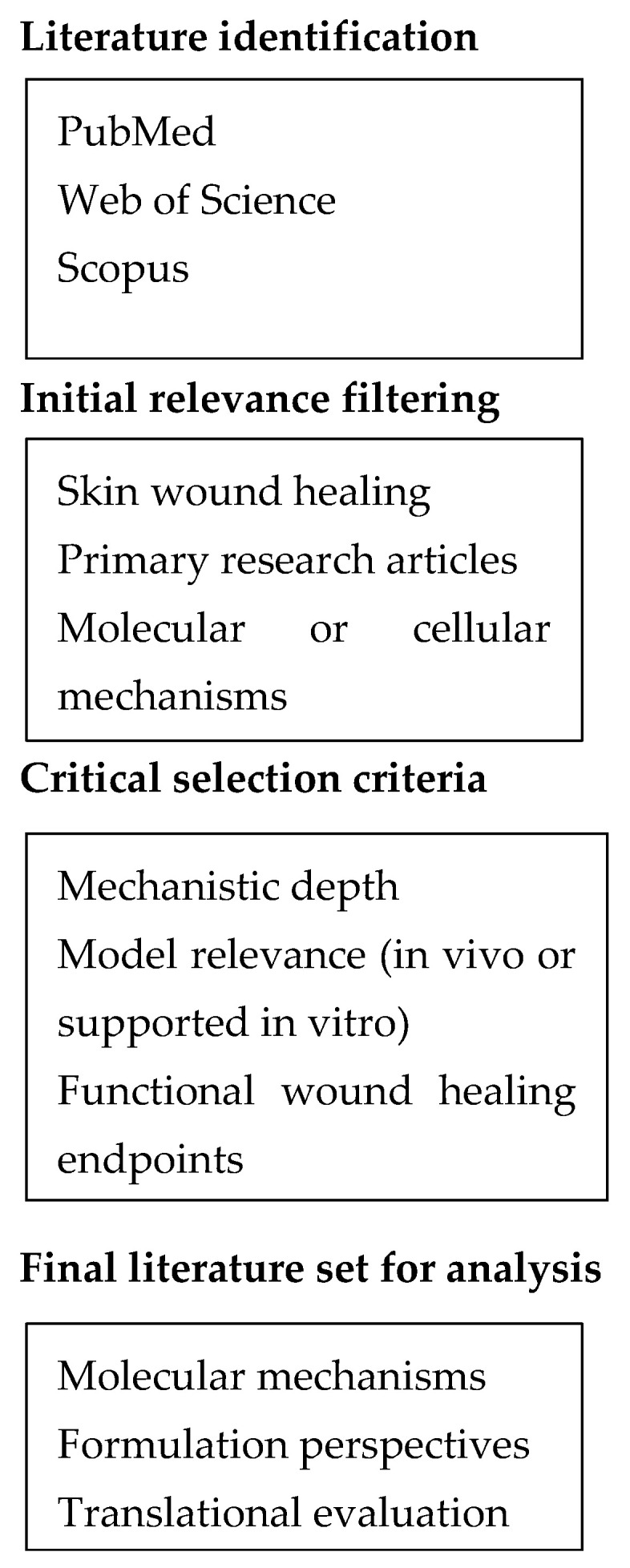
Conceptual overview of the literature identification and critical evaluation framework applied in this narrative review. The diagram illustrates the structured approach used to identify and integrate relevant primary research articles based on mechanistic relevance to molecular signalling pathways and functional outcomes in skin wound healing. The workflow represents a conceptual framework guiding literature integration rather than a formal systematic review process.

Inclusion criteria required that studies examined defined molecular pathways involved in inflammation, redox regulation, growth factor signalling, angiogenesis, or cell migration, and that they reported outcomes linked to tissue repair rather than wound closure alone. Purely descriptive studies, ethnopharmacological surveys, reviews, and opinion pieces were excluded. The retained literature was critically evaluated based on mechanistic depth, appropriateness of the experimental model, temporal and cell-type resolution, and the presence of functional healing endpoints. When formulation strategies were discussed, evaluation considered whether delivery systems were mechanistically justified, aligned with the targeted phase of wound healing, and supported by translationally relevant outcomes rather than by physicochemical performance alone. Because the objective of this work is to provide a mechanism-oriented synthesis rather than a formal systematic review, the selected literature was integrated through a structured narrative framework emphasizing mechanistic relevance, translational context, and functional outcomes related to skin repair. The literature identification and selection process used in this review is summarized in Figure 1.

## 3. Molecular Mechanisms of Skin Repair Modulated by Natural Extracts

This section synthesizes mechanistic evidence from primary preclinical studies addressing how plant-derived phytochemicals modulate key molecular pathways across distinct phases of skin wound healing. Rather than presenting biological outcomes in isolation, the evidence is organized according to defined signalling networks and chemically responsive cellular processes governing inflammatory resolution, redox homeostasis, angiogenesis, cellular migration, and tissue remodelling.

### 3.1. Modulation of Inflammatory Signalling Pathways

Inflammation is an essential component of wound repair; however, the magnitude and temporal coordination of inflammatory signalling determine whether early host defence is timely resolved to permit effective tissue regeneration. Excessive, prolonged, or poorly coordinated inflammatory activation may disrupt this transition and impair tissue repair. Plant-derived extracts influence this balance primarily through quantitative and context-dependent modulation of intracellular signalling cascades rather than through complete pathway inhibition [10,11].

Among the signalling pathways involved, the NF-κB axis plays a central regulatory role, as it governs early cytokine production, leukocyte recruitment, and oxidative stress responses. Evidence from multiple in vivo wound models indicates that structurally diverse phytochemical classes attenuate excessive NF-κB activation in wound tissue, accompanied by reductions in pro-inflammatory mediators such as TNF-α, IL-1β, and IL-6. Importantly, this attenuation does not appear to abolish early inflammatory signals required for debris clearance and antimicrobial defence. Instead, phytochemicals generally exert a modulatory effect that limits sustained or exaggerated activation. Nevertheless, direct temporal analyses examining early inflammatory kinetics remain limited [10,11].

This recalibrating effect is both context- and dose-dependent, and complete suppression of NF-κB signalling is neither observed nor desirable in physiologically relevant models of skin repair [12]. The extent of pathway modulation also appears to be influenced by the chemical class and structural features of the active compounds. Among polyphenolic compounds, flavonoids such as luteolin and structurally related molecules including quercetin have been associated with attenuation of NF-κB activation and downstream cytokine expression. Catechins, particularly epigallocatechin gallate (EGCG), exhibit concentration-dependent and structurally influenced modulation of inflammatory mediators, reflecting differences in hydroxylation patterns and gallate substitution. These findings support a modulatory, rather than suppressive, role of polyphenols in early inflammatory signalling and suggest that specific structural characteristics of phenolic scaffolds contribute to selective pathway engagement and transcriptional regulation [11,13].

Beyond NF-κB-mediated transcriptional control, upstream mitogen-activated protein kinase (MAPK) networks further shape inflammatory signalling dynamics during wound repair. MAPK pathways, including p38, JNK, and ERK, represent additional regulatory nodes through which natural compounds influence inflammatory responses. Stress-activated kinases such as p38 and JNK are frequently downregulated by flavonoids and phenolic acids, correlating with reduced production of inflammatory mediators, whereas ERK signalling, more closely associated with cell survival and proliferation, tends to be preserved or indirectly enhanced [12,14]. This differential regulation suggests that plant-derived bioactives rebalance inflammatory signalling toward a pro-healing phenotype rather than inducing nonspecific pathway inhibition. However, in many studies, MAPK modulation is inferred primarily from phosphorylation status, with limited integration of downstream transcriptional changes or functional wound healing endpoints. Moreover, most available data derive from keratinocyte and macrophage models, with comparatively limited validation in complex in vivo systems [14].

At the level of downstream inflammatory effectors, the cyclooxygenase-2 (COX-2) and inducible nitric oxide synthase (iNOS) axis represents another frequently reported molecular target of natural extracts. Terpenoids and triterpenes, in particular, have been shown to reduce COX-2 and iNOS expression in inflamed skin, thereby limiting prolonged prostaglandin and nitric oxide production associated with chronic inflammation [11,12]. Although these effects are often interpreted as beneficial, excessive emphasis on enzyme downregulation risks oversimplifying the context-dependent roles of COX-2 and iNOS, which also contribute to angiogenesis and extracellular matrix remodelling during later stages of the healing cascade. Consequently, critical evaluation increasingly focuses on whether COX-2 and iNOS modulation translates into improved tissue architecture and functional repair outcomes rather than solely reductions in inflammatory biomarkers [11].

At the functional cellular level, inflammatory modulation by natural extracts extends beyond pathway attenuation to immune phenotype reprogramming. Several studies indicate that specific phytochemicals facilitate the transition from a pro-inflammatory to a resolution phase by promoting macrophage polarization toward a resolution-associated M2-like phenotype and increasing IL-10 production [10,15]. These changes contribute to the establishment of a microenvironment conducive to fibroblast activation and re-epithelialization. Nonetheless, much of the supporting evidence remains correlative, particularly when derived from in vitro macrophage assays lacking in vivo validation. In selected models, attenuation of inflammatory signalling is accompanied by reduced microbial burden at the wound site, suggesting that immunomodulatory effects may indirectly enhance antimicrobial defence even in the absence of direct bactericidal activity [10,15].

Taken together, available evidence indicates that plant-derived phytochemicals do not function as broad-spectrum anti-inflammatory agents, but rather as selective modulators of interconnected signalling networks governing the initiation, amplification, and resolution of inflammatory responses during wound repair. The direction and magnitude of pathway engagement appear to depend on chemical class, structural features, concentration, and temporal context. This layered regulation highlights the importance of mechanistic resolution when evaluating natural extracts, as therapeutic benefit requires preservation of early host defence while preventing sustained inflammatory activation that compromises tissue regeneration.

### 3.2. Regulation of Growth Factor-Mediated Responses

Growth factor signalling functions as a central regulatory interface between inflammatory resolution and tissue regeneration during wound healing. Among the principal mediators, transforming growth factor-β (TGF-β) and vascular endothelial growth factor (VEGF) exert context-dependent and temporally sensitive effects on tissue repair. The magnitude, duration, and cellular localization of their activation determine whether regeneration proceeds toward functional restoration or maladaptive remodelling. Evidence from preclinical wound models indicates that plant-derived extracts influence these pathways primarily through modulation of downstream signalling dynamics rather than sustained growth factor overexpression.

Within this regulatory framework, TGF-β signalling plays a pivotal role in orchestrating fibroblast activation and extracellular matrix deposition. In fibroblasts, several triterpenoids and saponin-rich extracts enhance TGF-β/Smad2/3 signalling during the proliferative phase of healing, particularly at early to intermediate post-injury time points, where increased myofibroblast differentiation and matrix synthesis are observed [12,16]. Such activation appears beneficial when temporally restricted. In contrast, prolonged or excessive TGF-β signalling, especially in the absence of Smad7-mediated negative feedback, is associated with fibrotic remodelling and aberrant collagen accumulation rather than organized tissue restoration [2,16]. These findings emphasize that phytochemical engagement of TGF-β pathways must remain quantitatively and temporally controlled.

While TGF-β primarily governs matrix deposition and fibroblast differentiation, VEGF-dependent angiogenesis ensures the metabolic support required for regenerating tissue. VEGF signalling in endothelial cells is frequently enhanced in response to polyphenols and curcuminoids, either directly or indirectly through redox-sensitive stabilization of hypoxia-inducible factor-1α (HIF-1α) [17]. Contributions from fibroblast, and macrophage, derived VEGF further amplify these responses within the wound microenvironment. In vivo models demonstrate that moderate enhancement of VEGF signalling correlates with increased capillary density and accelerated wound closure [18]. However, angiogenic activity attributed to natural extracts is often inferred from surrogate endpoints such as VEGF expression levels or in vitro tube formation assays, which provide limited information regarding vessel maturation, perfusion efficiency, or long-term stability [19]. Accordingly, interpretation of angiogenic modulation requires integration of structural and functional vascular endpoints rather than reliance on molecular markers alone.

Importantly, the extent of growth factor modulation appears to be influenced by the chemical characteristics and concentration of the active phytochemicals. Polyphenols may indirectly regulate angiogenic responses through redox-sensitive signalling adjustments that affect HIF-1α stabilization and VEGF pathway activation. Curcuminoids, particularly curcumin, as well as selected flavonoids, have been reported to enhance endothelial migration and capillary formation in experimental wound systems; however, these effects remain strongly dependent on dose, timing, and the surrounding inflammatory context [17,20]. This variability features the importance of chemical structure and pharmacodynamic context in determining pathway engagement.

Beyond individual pathway effects, crosstalk between growth factor networks further shapes repair outcomes. TGF-β signalling can indirectly influence angiogenesis through paracrine interactions between fibroblasts and endothelial cells, while VEGF-driven neovascularization may reciprocally regulate fibroblast proliferation and collagen organization [21]. Certain plant-derived extracts appear to transiently coordinate these processes, fostering a pro-regenerative signalling environment that supports keratinocyte migration and re-epithelialization without sustaining profibrotic cues. Nonetheless, mechanistic evidence for coordinated pathway regulation remains limited, as many studies rely on single time-point analyses and lack cell-type-specific resolution within the wound microenvironment in vivo [21].

From a translational standpoint, modulation of growth factor signalling by natural extracts must be interpreted with caution. Acute wound models may overestimate regenerative benefits compared to chronic or metabolically impaired healing conditions in which baseline growth factor dysregulation is already present [5]. Moreover, variability in extract composition, phytochemical concentration, and dosing complicates reproducibility and risk assessment, particularly with respect to fibrotic outcomes [2].

Overall, current evidence indicates that plant-derived compounds can fine-tune growth factor-mediated signalling during skin repair through temporally restricted and context-dependent modulation of TGF-β and VEGF pathways. Establishing therapeutic relevance, however, requires temporally resolved and cell-type-specific investigations that directly link molecular pathway engagement to durable and functionally integrated healing outcomes [3].

### 3.3. Oxidative Stress Control and Redox Homeostasis

Redox homeostasis is a central determinant of phase-specific wound progression. Reactive oxygen species (ROS) function not only as cytotoxic by-products of inflammation but also as essential signalling mediators coordinating immune activation, cellular proliferation, angiogenesis, and matrix remodelling. The concentration, spatial distribution, and temporal resolution of ROS generation therefore dictate whether oxidative processes promote regenerative signalling or instead induce tissue damage. Within this adaptive framework, the nuclear factor erythroid 2–related factor 2 (Nrf2)–Kelch-like ECH-associated protein 1 (Keap1) axis serves as a principal regulatory mechanism through which cells respond to oxidative stress during cutaneous repair [22,23].

Plant-derived phytochemicals influence redox balance primarily through modulation of Nrf2-dependent transcriptional activity rather than through direct radical scavenging alone. Activation of Nrf2 and its subsequent nuclear translocation induces a coordinated transcriptional program enhancing endogenous antioxidant defences. Most supporting evidence derives from gene expression analyses and short-term functional assays demonstrating upregulation of canonical downstream targets such as heme oxygenase-1 (HO-1) and NAD(P)H quinone dehydrogenase 1 (NQO1) [22,23]. These effectors contribute to cytoprotection, redox buffering, and resolution of oxidative stress, particularly in keratinocytes and macrophages operating within the inflammatory wound microenvironment [13,24].

At the molecular level, several phytochemical classes, including flavonoids, phenolic acids, and selected terpenoids, engage the Nrf2–Keap1 system through redox-sensitive modification of cysteine residues on Keap1 or through indirect modulation of upstream kinases regulating Nrf2 stability [13,23]. In keratinocytes, Nrf2 activation has been associated with enhanced resistance to oxidative injury and improved migratory capacity, primarily in acute or in vitro stress models [4,13]. In fibroblasts, Nrf2-dependent redox regulation appears to support cellular survival and extracellular matrix synthesis during the proliferative phase of wound healing [22]. However, sustained or excessive activation of Nrf2 during later remodelling stages may disrupt redox-sensitive signalling required for coordinated growth factor responsiveness and matrix reorganization, accentuating the phase-dependent nature of this pathway [22,25].

A critical conceptual distinction in evaluating redox modulation concerns the difference between antioxidant capacity and regulation of redox signalling networks. Although many studies report reduced ROS levels following treatment with natural extracts, diminished ROS concentrations alone do not establish therapeutic benefit. Low-to-moderate ROS levels are required for angiogenic signalling, growth factor activation, and cellular migration, and indiscriminate suppression of ROS may therefore impair regenerative processes [25]. Mechanistic relevance is strongest in studies demonstrating that Nrf2-mediated transcriptional responses translate into functional repair outcomes, including improved wound closure rates, preservation of newly formed tissue integrity, or enhanced tensile strength, rather than solely reductions in biochemical markers of oxidative stress [4,25].

This distinction is particularly pertinent for polyphenolic compounds, which are frequently characterized as direct antioxidants. Emerging evidence suggests that their contribution to wound healing arises predominantly from modulation of redox-sensitive signalling pathways, especially Nrf2–Keap1 activation, rather than from stoichiometric radical neutralization. In this context, chemical structure, concentration, and cellular microenvironment collectively determine the direction and magnitude of redox pathway engagement [13,22].

Despite growing interest in redox-targeted mechanisms, several limitations temper translational interpretation. Much of the available evidence is derived from in vitro systems or short-term in vivo models relying on expression-based readouts of Nrf2 or its downstream targets, without assessing long-term scar architecture, biomechanical strength, or tissue organization [4,23]. In addition, insufficient resolution of cell-type-specific responses within the wound microenvironment complicates attribution of observed redox effects to keratinocytes, fibroblasts, endothelial cells, or immune populations [5].

To sum up, when considered within a redox-signalling framework, the available evidence indicates that plant-derived compounds support wound repair through context-dependent, Nrf2-mediated modulation of oxidative stress responses. Establishing durable therapeutic relevance, however, requires temporally resolved and cell-type-specific investigations that directly connect redox pathway engagement to structurally integrated and functionally meaningful repair outcomes [4].

### 3.4. Effects on Cellular Proliferation and Migration

Effective wound closure requires coordinated proliferation and migration of multiple skin-resident cell populations, including keratinocytes, fibroblasts, and endothelial cells. These processes must occur in a temporally regulated and spatially organized manner to restore epidermal continuity and dermal structure. Plant-derived compounds influence cellular dynamics primarily through modulation of intracellular signalling pathways governing cytoskeletal organization, cell-cycle progression, and cell–matrix interactions rather than through indiscriminate stimulation of cell growth [26,27].

In keratinocytes, several phytochemicals activate signalling cascades such as ERK1/2 and PI3K/Akt, pathways that regulate cytoskeletal reorganization, cell survival, and directional migration at the wound edge [26,27]. Notably, multiple polyphenolic compounds have been specifically implicated in promoting keratinocyte migration through activation of ERK1/2 and PI3K/Akt signalling axes. These effects are most consistently observed under oxidative or inflammatory stress conditions, where baseline cellular motility is impaired. This pattern suggests that polyphenols function primarily to restore compromised migratory capacity rather than to induce generalized or excessive proliferative responses. Enhanced re-epithelialization reported in preclinical models therefore appears to arise from improved coordination of migration and survival signalling rather than from supraphysiological stimulation of cell division [26].

Fibroblast responses to natural extracts are similarly context-dependent and reflect the dual requirement for cellular expansion and regulated extracellular matrix deposition. Phytochemical-mediated modulation of TGF-β-related signalling and focal adhesion pathways has been associated with enhanced fibroblast migration and matrix-producing activity in experimental wound systems [27,28]. However, increased fibroblast proliferation alone does not equate to improved healing outcomes. Excessive or poorly regulated fibroblast activation, particularly within TGF-β-enriched microenvironments, may predispose to fibrotic remodelling and disorganized collagen deposition [16]. Mechanistic relevance is therefore strongest in studies demonstrating that phytochemical-induced fibroblast migration and proliferation translate into improved granulation tissue architecture and orderly remodelling, rather than isolated increases in cell number [28].

Beyond keratinocytes and fibroblasts, endothelial cell proliferation and migration reinforce angiogenic sprouting and vascular network formation. Phytochemicals influence these processes indirectly through modulation of growth factor signalling and redox-sensitive pathways. In vitro assays such as scratch wound closure, transwell migration, and tube formation are frequently employed to demonstrate endothelial responses; however, these systems provide limited information regarding vessel maturation, perfusion efficiency, or long-term vascular stability. In vivo evidence is more compelling when enhanced endothelial activity is accompanied by histological or functional assessments confirming neovascular integration within regenerating tissue [29].

Across cell types, a recurring limitation in the literature is reliance on short-term or single-cell assays to infer regenerative benefit [30]. Proliferation markers or migration rates measured in isolation may overestimate therapeutic relevance if not contextualized within the multicellular and temporally regulated wound environment. In addition, dose dependence and extract composition are rarely standardized, complicating translational interpretation and reproducibility.

Within this framework, plant-derived compounds appear to support wound repair by facilitating coordinated and phase-appropriate regulation of cellular proliferation and migration across keratinocyte, fibroblast, and endothelial compartments. Therapeutic relevance depends on integration of these cellular responses within the broader sequence of tissue repair events rather than on isolated enhancement of proliferative or migratory indices [30].

To improve conceptual traceability between phytochemical classes, molecular targets, and phase-specific biological responses, the principal signalling pathways, representative phytochemical groups, primary cellular targets, and associated functional outcomes implicated in plant-mediated wound repair are summarized in Table 1 [31].

### 3.5. Polyphenols as Cross-Pathway Modulators

Polyphenols constitute one of the most extensively investigated phytochemical classes in wound healing, largely due to their capacity to interact with multiple redox-sensitive and growth-regulatory signalling networks. Rather than operating through a single dominant mechanism, polyphenolic compounds influence interconnected pathways governing inflammation, oxidative stress adaptation, angiogenesis, and cellular migration. Experimental evidence indicates that members of this class modulate NF-κB and MAPK signalling during inflammatory resolution, regulate Nrf2-dependent antioxidant responses, and influence VEGF-mediated angiogenic activity in a context-dependent manner [20].

This cross-pathway engagement reflects the structural characteristics of polyphenols, including multiple hydroxyl groups and conjugated aromatic systems, which enable redox-sensitive interactions and modulation of signalling proteins. However, biological activity is strongly influenced by concentration, temporal exposure, and formulation-dependent bioavailability. Consequently, identical compounds may exhibit pro-resolving, neutral, or even inhibitory effects depending on experimental context and dosing parameters, precluding generalized assumptions regarding therapeutic benefit [20,23].

A recurring limitation in the literature is the reliance on surrogate molecular readouts, such as reduced cytokine expression or increased antioxidant enzyme levels, without parallel assessment of structural tissue organization, biomechanical strength, or long-term remodelling outcomes [13,20,22]. As a result, the designation of polyphenols as broadly “regenerative” agents often exceeds the available functional evidence.

Accordingly, interpretation of polyphenol-mediated effects in wound repair requires integration of chemical structure, pathway engagement, temporal dynamics, and tissue-level outcomes. Only through such mechanistic and functional alignment can their true therapeutic relevance be established.

## 4. Evidence from Key Medicinal Plants

To avoid a purely descriptive botanical overview, the following representative plants are analyzed with emphasis on their dominant phytochemical classes and mechanistic characteristics within the wound-healing cascade [32]. Because cultivation conditions, harvest timing, plant part selection, and extraction methodologies directly influence phytochemical composition, they also influence the observed mechanistic effects of plant extracts. Consequently, the lack of consistent extract standardization and complete chemical characterization across the literature represents a significant limitation when interpreting and comparing reported mechanistic findings.

Across the cited preclinical literature, preparations most commonly include aqueous or ethanolic extracts or chemically enriched fractions. Representative examples include polysaccharide-rich leaf gel from *Aloe vera*, triterpenoid-enriched aerial extracts from *Centella asiatica*, curcuminoid-rich rhizome fractions from *Curcuma longa*, flavonoid- and triterpenoid-containing flower extracts from *Calendula officinalis*, and ginsenoside-rich root extracts from *Panax ginseng*. These compositional differences must be considered when attributing pathway engagement to specific phytochemical classes and when comparing biological outcomes across studies.

Despite substantial chemical diversity, reported wound-healing effects converge mechanistically on a restricted set of molecular and cellular processes regulating inflammatory resolution, redox homeostasis, angiogenesis, and extracellular matrix remodelling. Among the most extensively studied botanical sources, *Aloe vera*, *Centella asiatica*, *Curcuma longa*, *Calendula officinalis*, and *Panax ginseng* provide representative examples of how distinct phytochemical profiles intersect with shared regulatory pathways, albeit with varying degrees of mechanistic clarity and translational maturity.

Accordingly, the following subsections examine these species in relation to their predominant phytochemical constituents and experimentally supported engagement across defined phases of tissue repair.

### 4.1. Aloe vera

Preparations derived from the inner leaf gel of *Aloe vera* are enriched in acetylated polysaccharides, glycoproteins, and low-molecular-weight phenolic constituents [33]. Preclinical wound models report attenuation of sustained NF-κB activation during early inflammatory phases, accompanied by enhanced keratinocyte migration under oxidative stress conditions. These effects are consistent with modulation of Nrf2-dependent antioxidant pathways and restoration of redox homeostasis as discussed in Section 3.3 [33].

However, extract heterogeneity and limited chemical standardization complicate the attribution of observed effects to specific molecular constituents. While topical formulations containing *Aloe vera* demonstrate supportive wound care properties, evidence for selective, pathway-specific modulation remains comparatively limited. Moreover, its reported antimicrobial activity appears to be largely indirect, arising from improved barrier restoration and modulation of local immune responses rather than from direct bactericidal action [33].

### 4.2. Centella asiatica 

Aerial extracts *of Centella asiatica* derived from leaves, contain triterpenoid saponins, notably asiaticoside and madecassoside, which represent the principal bioactive constituents [34]. These compounds consistently engage TGF-β/Smad signalling in fibroblasts, promoting granulation tissue formation and collagen deposition in experimental wound systems [34,35]. Upregulation of matrix-associated genes and increased myofibroblast differentiation have been reported at early proliferative stages.

Nevertheless, TGF-β pathway engagement is dose-dependent and temporally sensitive. Sustained activation may predispose to excessive collagen accumulation and fibrotic remodelling, particularly when extracts are incorporated into delivery systems that enhance local exposure [35]. Although mechanistic resolution is comparatively strong relative to other botanicals, long-term scar architecture and biomechanical outcomes remain insufficiently characterized.

### 4.3. Curcuma longa

Curcuminoids represent a structurally distinct subclass of polyphenolic compounds that function as cross-pathway modulators in wound repair [20]. Derived primarily from rhizome extracts of *Curcuma longa*, curcuminoids constitute the principal bioactive fraction and are characterized by conjugated diketone structures that enable redox-sensitive and transcriptional regulatory interactions. Mechanistically, these compounds intersect multiple signalling axes, including NF-κB-mediated inflammatory pathways, Nrf2-dependent redox adaptation, and VEGF-associated angiogenic responses, thereby linking inflammatory modulation, oxidative stress control, and vascular remodelling within a unified regulatory framework [20].

Consistent with the pathway architecture outlined in Section 3.1 and Section 3.3, curcuminoids attenuate pro-inflammatory transcriptional activity, enhance antioxidant defence mechanisms, and indirectly modulate hypoxia-responsive VEGF signalling under stress conditions. While such multimodal activity is mechanistically attractive, translational interpretation remains constrained by poor intrinsic aqueous solubility and limited bioavailability. As a result, many reported therapeutic effects depend heavily on formulation-driven enhancement strategies, including nanoparticle systems and lipid-based carriers (see Section 5) [20]. Consequently, attribution of biological efficacy to the botanical extract itself is often inseparable from the employed delivery system.

In selected preclinical models, curcuminoid-based interventions have been associated with improved collagen fibre organization and enhanced tensile strength of regenerated tissue. However, these outcomes appear to depend on tightly regulated growth factor dynamics rather than indiscriminate stimulation of matrix deposition, underscoring the importance of dose- and phase-dependent pathway modulation.

### 4.4. Calendula officinalis

Extracts of *Calendula officinalis*, most commonly derived from flower material, contain flavonoids, triterpenoids, carotenoids, and volatile constituents. The literature predominantly attributes anti-inflammatory and antimicrobial properties to these preparations, with additional reports of enhanced fibroblast migration and early angiogenic signalling [36]. However, the majority of available studies emphasize phenotypic endpoints, such as accelerated wound closure, without detailed mechanistic correlation to defined molecular pathways.

As a consequence, *Calendula officinalis* occupies an intermediate evidentiary position. Although consistent supportive effects on wound repair are reported, mechanistic under-characterization limits precise attribution of pathway engagement relative to more extensively studied species such as *Centella asiatica* or *Curcuma longa* [36]. Reported antimicrobial activity is similarly described largely at a phenotypic level and rarely linked to defined molecular targets, reducing translational specificity despite reproducible observational outcomes.

### 4.5. Panax ginseng

*Panax ginseng* provides a distinct mechanistic profile mediated primarily by ginsenosides present in root-derived extracts [37]. These triterpenoid saponins have been reported to influence angiogenesis, cellular proliferation, and redox-sensitive signalling pathways in preclinical wound models. Coordinated effects on endothelial migration, fibroblast activation, and VEGF-associated signalling cascades suggest engagement of pro-regenerative networks integrating vascular and stromal responses [37].

Despite promising mechanistic observations, translational advancement remains constrained by variability in ginsenoside composition across preparations and insufficient chemical standardization. Moreover, limited comparative studies against structurally simpler botanical extracts or synthetic pathway modulators restrict definitive assessment of relative efficacy [18,37]. Greater chemical resolution and dose–response characterization is therefore required to define reproducible therapeutic parameters.

### 4.6. Overall Mechanistic Considerations

These botanical examples demonstrate that therapeutic relevance in wound healing is not determined by botanical identity per se, but by the alignment of defined phytochemical composition, specific molecular pathway engagement, and formulation-dependent bioavailability with temporally regulated and phase-appropriate modulation of tissue repair processes. Therapeutic efficacy therefore emerges from controlled interaction between chemical composition and biological context rather than from plant origin alone.

However, this alignment is frequently obscured by methodological variability across studies. Extrapolation of preclinical findings often occurs without rigorous control of dose, exposure duration, extract standardization, and chemical composition [32]. Because phytochemical profiles vary according to cultivation, harvesting, and extraction parameters, incomplete structural characterization limits reproducibility, complicates cross-study comparison, and weakens definitive mechanistic attribution of observed effects.

Consequently, meaningful progress in this field will depend less on expanding the catalog of candidate plants and more on integrating standardized phytochemical profiling, structure–activity analysis, pathway-specific mechanistic validation, and functionally relevant outcome assessment within translationally oriented evaluation frameworks [32]. Only through such chemically and mechanistically aligned approaches can plant-derived extracts be positioned as reproducible and molecularly defined therapeutic candidates in wound repair.

## 5. Dermopharmaceutical and Formulation Perspectives

As discussed above, preclinical investigations provide substantial mechanistic evidence supporting the wound-healing potential of plant-derived extracts. However, translating these findings into clinically applicable dermopharmaceutical products remains challenging, primarily due to formulation-related physicochemical and biopharmaceutical limitations. Specifically, many wound-relevant phytochemicals exhibit poor aqueous solubility, chemical instability under physiological conditions, and limited cutaneous permeability factors that collectively restrict effective local bioavailability at the wound site [38].

Beyond these intrinsic limitations, the compositional complexity and batch-to-batch variability of botanical extracts further complicate dose standardization, reproducibility, and therapeutic predictability [32]. Heterogeneous phytochemical profiles and incomplete quantitative characterization often impede robust correlations between chemical composition and observed biological effects.

Therefore, selection of dermopharmaceutical delivery systems should be guided by the solubility, stability, and permeability characteristics of the dominant phytoconstituents. Poor aqueous solubility, oxidative instability, and limited tissue penetration frequently necessitate encapsulation strategies or carrier-based systems designed to enhance local exposure while preserving chemical integrity [38]. Importantly, most advanced delivery platforms primarily address pharmacokinetic constraints rather than enhance intrinsic biological activity.

Firstly, hydrogel-based systems are among the most extensively studied platforms in preclinical wound models, owing to their capacity to maintain hydration, enable sustained release, and retain hydrophilic or macromolecular constituents at the wound interface [39]. These systems are particularly compatible with hydrophilic or polysaccharide-rich extracts, such as those derived from *Aloe vera*, where hydration support aligns with the physicochemical properties of the principal bioactive components. Hydrogels may also modulate microenvironmental parameters, including hydration, pH, and oxygen diffusion, there by indirectly supporting cellular migration and extracellular matrix remodelling [38]. Nevertheless, their therapeutic contribution is largely supportive, as reported benefits may partially reflect carrier-mediated effects rather than direct pharmacodynamic enhancement [39].

Secondary, lipid-based carriers, including nanoemulsions, liposomes, and solid lipid nanoparticles, are primarily investigated to improve the solubility, stability, and cutaneous delivery of lipophilic phytochemicals such as terpenoids and curcuminoids [38,40]. These systems are particularly relevant for poorly water-soluble constituents, including curcuminoids from *Curcuma longa* and selected terpenoid compounds present in botanical extracts. In preclinical models, lipid-based formulations have been shown to increase local deposition and prolong residence time within superficial skin layers, effects that may be most relevant during the early inflammatory phase of repair [40].

Despite these pharmacokinetic improvements, enhanced penetration does not necessarily translate into superior functional outcomes, particularly when clinically meaningful endpoints, such as tensile strength, scar architecture, or long-term tissue integrity, are not evaluated [40]. Formulation studies may conflate technological sophistication with therapeutic advancement, and claims that nanocarriers intrinsically enhance wound repair are often insufficiently substantiated when compared with simpler formulations or appropriate controls. In many cases, nanoformulation strategies developed for compounds such as curcumin or ginsenosides primarily overcome solubility and stability constraints rather than amplifying pathway-specific biological effects [41].

Moreover, enhanced bioavailability does not inherently improve therapeutic precision. Increased exposure may introduce unintended consequences, including off-target signalling or excessive activation of pathways such as TGF-β, potentially elevating fibrotic risk [16]. Accordingly, mechanistic alignment between the delivery platform, chemical properties of the constituents, and the targeted wound-healing phase remains critical yet frequently underdeveloped [41].

Beyond mechanistic considerations, translation into regulated dermopharmaceutical products introduces additional challenges, including scalability, long-term nanomaterial safety, and regulatory classification of botanical formulations [40,42]. Regulatory authorities require well-defined compositional, stability, and safety data, particularly under pharmaceutical regulatory frameworks [42].

Overall, the translational value of dermopharmaceutical formulations depends less on technological sophistication than on rational, mechanism-guided design coupled with rigorous evaluation of functional healing outcomes and regulatory feasibility [29,39]. Enhanced bioavailability or penetration does not inherently confer therapeutic advantage, particularly when pathway activation exceeds the narrow thresholds required for regenerative rather than fibrotic repair [16].

Accordingly, formulation strategies must be aligned simultaneously with (i) the physicochemical properties of the dominant phytoconstituents, (ii) the targeted molecular pathways, and (iii) the specific phase of wound healing in which modulation is intended. Hydrophilic and polysaccharide-rich extracts (e.g., *Aloe vera*) are typically suited to hydrogel-based systems that support moisture balance and local retention, whereas poorly soluble lipophilic constituents, including curcuminoids (*Curcuma longa*) and ginsenosides (*Panax ginseng*), often require lipid-based or nanoscale carriers to achieve adequate local exposure [38,40].

Within this integrative framework, dermopharmaceutical platforms function primarily as modulators of exposure and spatial–temporal control rather than intrinsic amplifiers of biological activity. The principal formulation strategies, their mechanistic rationale, and key translational constraints are summarized in Table 2 [39,40].

## 6. Limitations, Knowledge Gaps, and Translational Challenges

Beyond the formulation-related and regulatory considerations discussed above, additional conceptual and methodological limitations continue to constrain interpretation and translational advancement in the field. A major limitation concerns the frequent oversimplification of extract activity without adequate resolution of the relative contributions of specific phytochemical subclasses, particularly polyphenols [36]. Although substantial progress has been made in delineating how plant-derived extracts modulate wound-healing pathways, several systemic constraints persist.

A recurring limitation across mechanistic studies is the reliance on simplified experimental models that fail to recapitulate the temporal, multicellular, and phase-dependent organization of skin repair [43]. Pathway-modulation-based readouts therefore provide limited insight into how signalling events are dynamically coordinated across successive phases of healing [31,43]. Moreover, mechanistic conclusions are often extrapolated from in vitro or acute in vivo models without sufficient validation in chronic or impaired wound settings, where dysregulated inflammation, oxidative stress, and altered growth factor signalling fundamentally modify therapeutic responsiveness [5,43]. Such extrapolation may obscure context-dependent structure–activity relationships and concentration-dependent effects that are critical for therapeutic precision.

In parallel with these experimental limitations, intrinsic heterogeneity at the level of botanical material and extract composition represents a central and unresolved challenge. Variability in plant source, processing methods, extract composition, and dosing complicates cross-study comparisons and undermines reproducibility, even when nominally identical species are examined [32]. While advanced delivery systems may mitigate certain bioavailability and stability limitations, they also introduce additional layers of complexity, including altered pharmacodynamics, potential off-target effects, and regulatory uncertainty [40]. Importantly, improvements in penetration or local concentration do not inherently translate into superior healing outcomes, particularly when pathway activation exceeds the narrow therapeutic window required for regenerative rather than fibrotic responses [40]. Collectively, these factors highlight a persistent gap between mechanistic plausibility and functionally meaningful tissue repair [43].

These preclinical and material-level constraints are further compounded in the clinical setting by the multifactorial nature of chronic wounds and the absence of standardized evaluation frameworks. Many preclinical studies lack clinically relevant comparator treatments, and direct comparisons with established standard wound therapies are frequently absent, complicating assessment of the relative therapeutic value of plant-derived interventions [36]. Endpoints such as accelerated wound closure are often prioritized over long-term measures of tissue quality, tensile strength, or scar architecture, thereby limiting clinical relevance [43]. Furthermore, regulatory requirements for botanical-based dermopharmaceuticals demand well-defined composition, safety documentation, and manufacturing consistency—criteria that are particularly challenging to fulfil for multicomponent natural extracts incorporated into complex delivery systems [42]. Comprehensive physicochemical characterization, batch-to-batch consistency, and validated analytical control strategies are therefore essential for regulatory alignment.

Addressing these limitations will require a shift toward temporally resolved, cell-specific, and outcome-oriented study designs, combined with greater emphasis on extract standardization and comparative benchmarking [31,32]. Such an approach is necessary to advance the field beyond isolated proof-of-concept observations toward reproducible, mechanistically grounded, and clinically translatable wound-healing strategies [43].

## 7. Future Perspectives and Research Directions

Progress in the development of plant-derived therapeutics for skin repair will depend less on expanding candidate lists and more on the implementation of mechanism-guided and chemically resolved experimental design. Priority should shift from crude extracts toward active fractions, chemically defined phytochemical combinations, or single lead compounds with clearly characterized molecular targets [44]. Such an approach enables rigorous evaluation of dose–response relationships, temporal activity windows, and pathway-specific effects, particularly for signalling axes with narrow therapeutic ranges, including TGF-β-mediated matrix deposition and redox-sensitive Nrf2 signalling [2,16]. Mechanistic investigations must further resolve cell-type-specific responses within the wound microenvironment, recognizing that keratinocytes, fibroblasts, endothelial cells, and immune cells may exhibit divergent responses to the same bioactive stimulus [1,3,5].

Temporally resolved and functionally anchored study designs are required to capture the dynamic and phase-dependent progression of wound healing [31]. Single time-point measurements or isolated expression-based readouts rarely provide sufficient evidence of therapeutic relevance. Instead, pathway modulation should be correlated with integrated functional outcomes, including tissue architecture, tensile strength, vascular maturation, and scar quality, evaluated across distinct healing phases [31]. Increased reliance on chronic and impaired wound models is essential to determine whether proposed mechanisms remain operative under clinically relevant pathological conditions characterized by persistent inflammation, oxidative imbalance, and altered growth factor signalling [5,43].

From a translational perspective, formulation strategies should be guided by biological phase, target pathway, and physicochemical compatibility rather than technological complexity alone [40,41]. Delivery systems must be rationally aligned with the intended molecular target, cellular compartment, and wound-healing stage, ensuring mechanistic coherence between compound properties and therapeutic objectives. Concurrent efforts in chemical standardization, quantitative phytochemical profiling, comparative benchmarking against established standard-of-care therapies, and regulatory-aligned study design will be required to bridge preclinical findings with clinical feasibility [32,42]. Integrating comparative benchmarking into future research frameworks will be particularly important to contextualize mechanistic advantages within clinically meaningful performance thresholds.

Collectively, these considerations support a more disciplined integration of natural product chemistry, wound biology, and pharmaceutical formulation science. Such an approach positions plant-derived compounds as chemically characterized, mechanistically defined, and clinically contextualized therapeutic candidates, rather than broadly acting or empirically applied remedies [44]. An additional priority for future research involves the integration of mechanistic phytochemical research with standardized translational evaluation frameworks. Greater alignment between phytochemical characterization, pathway-specific mechanistic validation, and clinically relevant outcome measures will be necessary to ensure reproducibility and facilitate regulatory translation of plant-derived therapeutics. Establishing such integrative research strategies may help bridge the current gap between promising preclinical observations and the development of reliable dermopharmaceutical interventions for wound management.

## 8. Conclusions

The therapeutic relevance of plant-derived extracts in skin repair depends less on the breadth of reported bioactivities than on the precision with which specific molecular pathways are modulated within defined temporal windows of the healing process. Anti-inflammatory, redox-regulating, angiogenic, and pro-migratory effects contribute meaningfully to repair only when quantitatively controlled and mechanistically aligned with the phase-dependent biology of the wound and constrained within narrow functional thresholds. Uncontrolled pathway activation, including excessive inflammatory suppression, dysregulated growth factor signalling, or imbalanced redox modulation, carries a clear risk of impaired tissue quality or fibrotic remodelling, reinforcing the importance of pathway-specific and concentration-dependent modulation over nonspecific bioactivity claims.

Pharmaceutical relevance emerges at the intersection of molecular mechanism, formulation design, and translational feasibility. Advances in delivery systems can mitigate intrinsic physicochemical and bioavailability limitations of phytochemicals but do not replace the requirement for chemical characterization, mechanistic resolution, and functional outcome validation. Botanical complexity, extract variability, and regulatory constraints limit interpretability when descriptive outcomes are not supported by standardized chemical profiling and outcome-oriented evaluation frameworks. Within this framework, natural extracts are best viewed not as inherently regenerative agents, but as sources of chemically defined bioactive modulators whose therapeutic value depends on controlled, context-appropriate, and evidence-based integration into wound-healing strategies.

Future progress will depend on integrating advanced analytical characterization, quantitative structure–activity assessment, and standardized compositional control to ensure reproducibility, regulatory compliance, and clinically meaningful translation. By integrating phytochemical diversity, molecular signalling mechanisms, and pharmaceutical formulation considerations, the present review provides a structured framework for interpreting plant-derived extracts as mechanistically defined therapeutic candidates in skin repair research.

## Figures and Tables

**Table 1:** Molecular mechanisms targeted by plant-derived phytochemicals during distinct phases of skin wound healing.

Wound Healing Phase	Molecular Pathway/Axis	Representative Phytochemical Classes	Primary Cellular Targets	Functional Outcome in Wound Repair
Early inflammatory phase	NF-κB signalling	Flavonoids, phenolic acids, terpenoids	Keratinocytes, macrophages	Modulation of inflammatory cytokine production
Early inflammatory phase	MAPK pathways (p38, JNK, ERK)	Flavonoids, phenolic acids	Keratinocytes, macrophages	Regulation of inflammatory signalling dynamics
Resolution/proliferative transition	Macrophage polarization (M1 → M2-like)	Polyphenols, triterpenoids	Macrophages	Promotion of pro-resolving immune phenotype
Proliferative phase	TGF-β/Smad signalling	Triterpenoids, saponins	Fibroblasts	Fibroblast activation and matrix deposition
Proliferative phase	VEGF-mediated angiogenic signalling	Polyphenols, curcuminoids	Endothelial cells (direct); fibroblasts/macrophages (indirect)	Angiogenesis and neovascularization
Multiple phases	Nrf2–Keap1 redox signalling	Flavonoids, phenolic acids, terpenoids	Keratinocytes, fibroblasts, macrophages	Redox homeostasis and cytoprotection
Re-epithelialization	ERK1/2, PI3K/Akt	Diverse phytochemicals	Keratinocytes	Keratinocyte migration and wound closure

Mechanistic effects summarized in this table are derived from preclinical in vivo and mechanistically supported in vitro wound healing models. Reported pathway modulation reflects context and phase dependent observations and should not be interpreted as uniform or sustained activation across all stages of skin repair.

**Table 2:** Dermopharmaceutical formulation strategies for plant-derived wound healing agents: mechanistic rationale and translational constraints.

Formulation Type	Primary Delivery Rationale	Relevant Phytochemical Classes	Mechanistic Implications	Translational and Regulatory Limitations
Hydrogels	Local retention; moist wound environment; sustained release	Polysaccharides, hydrophilic polyphenols	Indirect support of cell migration and matrix remodelling via microenvironment modulation	Many benefits attributable to carrier properties rather than extract pharmacodynamics
Nanoemulsions	Improved solubility and dispersion of lipophilic compounds	Terpenoids, essential oils, curcuminoids	Increased local exposure in superficial wound layers, potentially enhancing early-phase signalling	Penetration metrics often not linked to improved tissue quality or long-term outcomes
Liposomes	Encapsulation and protection of unstable phytochemicals	Polyphenols, flavonoids	Altered cellular uptake and pathway engagement	Stability, scalability, and batch reproducibility remain challenging
Solid lipid nanoparticles	Controlled release and improved stability	Lipophilic phytochemicals	Prolonged residence time at the wound surface	Risk of overexposure and off-target pathway activation
Composite systems	Combination of retention and enhanced delivery	Mixed phytochemical profiles	Multimodal modulation of inflammatory and redox pathways	Increased formulation complexity complicates regulatory approval
Conventional topical formulations	Simplicity and regulatory familiarity	Broad	Supportive delivery without targeted modulation	Limited control over exposure and pathway specificity

Reported mechanistic implications are based on preclinical wound healing studies and reflect the combined contribution of phytochemical activity and delivery system associated effects. Improvements in formulation performance do not necessarily correspond to enhanced intrinsic bioactivity or clinically translatable therapeutic benefit.

## Data Availability

No new data were created or analyzed in this study. Data sharing is not applicable to this article.
